# Reactive Peri-Arthroplasty Pseudotumors: A Rare Cause of Hip Pain and Iatrogenic Sciatica

**DOI:** 10.7759/cureus.47322

**Published:** 2023-10-19

**Authors:** Adham Ahmed, Yisroel Grabie, Jeffrey Loeffler, Yosef Buchen, Sudeep Acharya

**Affiliations:** 1 Internal Medicine, City University of New York School of Medicine, New York, USA; 2 Internal Medicine, Staten Island University Hospital-Northwell Health, New York, USA; 3 Pulmonary and Critical Care Medicine, Staten Island University Hospital-Northwell Health, New York, USA

**Keywords:** sciatic nerve injury, metal-on-metal hip, prosthetic joint, pseudotumor cyst, total hip arthroplasty (tha)

## Abstract

Total hip arthroplasty (THA) is the preferred treatment for patients with hip joint disorders refractory to conservative management. While original implants were designed to articulate a metallic femoral head onto a polyethylene liner, the popularity of “metal-on-metal” (MoM) hip implants surged in the early 21st century due to their perceived superior long-term durability and lower revision rates. However, subsequent follow-up studies showed high failure rates due to inflammatory responses to periprosthetic metallic debris leading to lymphocytic proliferation, soft tissue necrosis or fibrosis, systemic metal toxicity, and/or the development of cystic pseudotumors. Although these discoveries resulted in a significant decrease in MoM THA and revision procedures, the majority of MoM hip implants persist in the adult population. In this case report and review, we report the presentation, diagnostic work-up, and management of an 84-year-old status-post MoM THA who presented with unilateral leg tenderness and poor ambulation secondary to pseudotumor-induced sciatica.

## Introduction

For individuals with hip disorders refractory to conservative management, surgical total hip arthroplasty (THA) is indicated for pain relief, increased ambulation, and improved quality of life [1]. Estimates project that over two million Americans are currently living with a THA, with an expected rise in the coming years due to an aging population [2]. The earliest model for hip implants articulated a metallic femoral head with a polyethylene liner (metal-on-polyethylene (MoP)), a design that was built upon by the introduction of cross-linked polyethylene, which facilitated improved long-term durability [3,4]. Despite these benefits, MoP implants are susceptible to aseptic loosening over time with subsequent degenerative osteolysis of the acetabular and femoral components due to progressive wear and tear [5]. Notably, while the progression of osteolysis secondary to polyethylene wear may be slowed down using non-steroidal anti-inflammatory drugs (NSAIDs) and bisphosphonate therapy, definitive treatment often necessitates a revision operation [6].

Alternatively, articulating a metal femoral head directly with a smooth metalloid acetabular cup (metal-on-metal (MoM)) was theorized to decrease long-term osteolysis implant failure compared to polyethylene liners while simultaneously providing increased stability and allowing larger femoral head sizes to be implanted [7]. However, long-term registry data for MoM was suboptimal, showing high failure rates and the development of adverse reactions to metal debris (ARMDs), including lymphocytic periprosthetic inflammation, metallosis due to metallic debris deposition in soft tissue, and the possible development of cystic pseudotumors secondary to the inflammation [8]. Furthermore, reports have described the development of systemic metal toxicity from the use of cobalt-chromium implants [9].

In light of these reports, the rates of MoM implants have dramatically decreased, now accounting for less than 1% of all THAs. Despite this, it is estimated that nearly 80% of MoM THAs implanted in the early 21st century remain in situ, meaning healthcare providers will inevitably be tasked with the diagnosis and management of patients presenting with ARMDs [10]. We report our experience managing an elderly man post-MoM chromium-cobalt THA 19 years ago who presented to our hospital with unilateral leg tenderness and poor ambulation secondary to a MoM pseudotumor with sciatic nerve involvement.

## Case presentation

**Figure 1:**
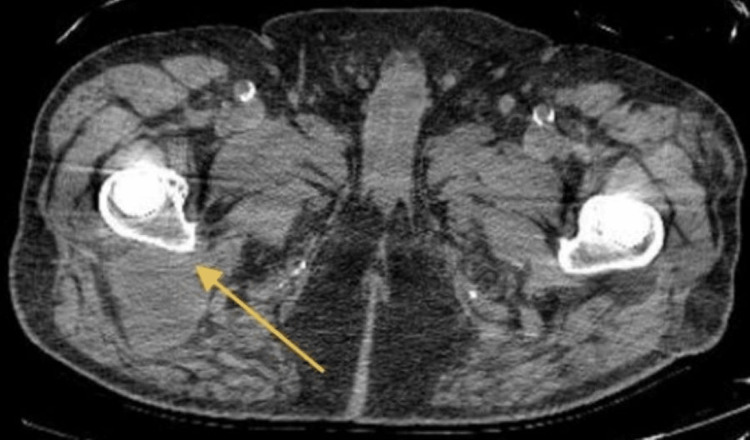
CT pelvis Fluid is present in lesser trochanteric bursa bilaterally. Large 5.2 x 5 centimeter fluid build-up in the right lesser trochanteric bursa.

The patient, an 84-year-old man with a past surgical history of bilateral THA (19 years ago), arrived at the emergency room complaining of significant acute-onset right hip pain. He had a medical history of nephrolithiasis, coronary artery disease, type 2 diabetes mellitus treated with metformin, and thrombocytopenia. The patient reported he was rising from a chair in the morning when the pain began and became worse with hip flexion and ambulation. He endorsed difficulty weight-bearing on the right side but can ambulate without assistance. He denied any fevers, chills, angina, dyspnea, hematuria, abdominal or flank pain, or gastrointestinal disturbances. The patient was admitted to the medicine service and underwent a formal musculoskeletal evaluation, including a pelvic X-ray, which ruled out a fracture, and a CT scan, which was significant for fluid in the bilateral lesser trochanteric bursa, slightly increased compared to a prior study from four years prior. The fluid measured up to 5.2 x 5 centimeters within the right lesser trochanteric bursa and was suggestive of bursitis (Figure 1).

Initial labs showed a normal white blood cell count of 6.55 K/uL (reference range: 4.80-10.80 K/uL), an elevated erythrocyte sedimentation rate of 128 mm/Hr (reference range: 0-10 mm/Hr), an elevated C-reactive protein of 11.4 mg/L (reference range: <4.0 mg/L), and a platelet count of 88 K/uL (reference range: 130-400 K/uL). Admission vital signs showed a temperature of 97.7 F, a heart rate of 69/min, a respiratory rate of 16/min, a blood pressure of 137/62 mm Hg, and an oxygen saturation of 97% on room air. A physical exam on admission revealed tenderness to palpation along the lateral right hip and groin, without rash, erythema, swelling, ecchymosis, or petechia. Given the lack of systemic symptoms, inflammatory markers, and alarming physical exam, there was a low suspicion of prosthetic joint infection (PJI), and the patient was recommended for outpatient follow-up with stretching exercises and NSAIDs as needed for pain.

**Figure 2:**
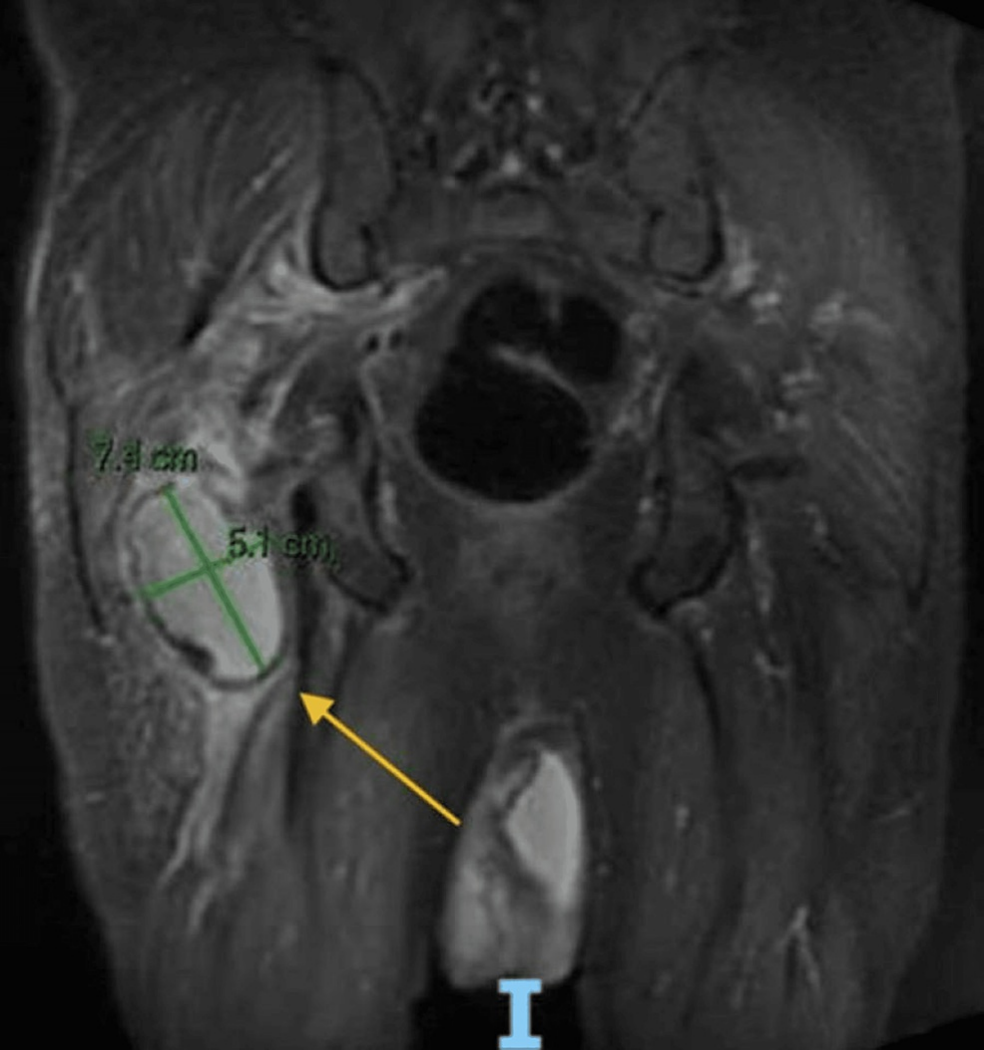
MRI pelvis MRI showing bilateral periprosthetic osteolysis is worse on the right side. A pseudotumor is noted on the right side, exerting a mass effect on the sciatic nerve at the level of the ischial tuberosity.

Over the next several hours, the patient’s pain continued to worsen, and he required morphine intravenously. During a physical therapy session, he ambulated 10 feet with a rolling walker before the pain forced him to stop. The pain management service was consulted, which recommended conservative management and an MRI of the hip or pelvis to assess ischeo-femoral impingement, which could be managed with an outpatient steroid injection. On hospital day three, an MRI of the right hip revealed well-demarcated osteolysis of the right hip arthroplasty femoral and acetabular components, most significant in the right anterior/inferior acetabulum and pubic root, and in the right greater trochanter. Of interest, the right sciatic nerve was compressed at the level of the ischial tuberosity by a pseudotumor (7.5 x 5 x 3 cm) on the posterior aspect of the proximal femur and a 6 x 4 x 2 cm pseudotumor at the superior margin of the acetabulum. Mildly localized muscular edema surrounded the right hip (Figure 2).

Two days later, the patient underwent percutaneous ultra-sound-guided drainage of the right posterior hip with aspiration of approximately 30 mL of bloody fluid. The patient tolerated the procedure well, and the fluid was taken for microbiological analysis. Fluid culture was significant for Lactobacillus species with numerous polymorphonuclear cells. An infectious disease consult was placed. At the time, we did not have access to microbial cell counts, which made it difficult to confidently rule out true PJI. However, antibiotic therapy was held due to the lack of systemic infectious symptoms, with a plan to repeat aspiration in the future with cell count and alpha-defensin testing. The leading theory for the development of the pseudotumor cyst was the suspected leak of metallic ions from his hip prosthetics, causing a periprosthetic inflammatory response. Of note, we ruled out systemic metal toxicity as the blood draw returned normal levels of cobalt (<1.0 microgram/L) and chromium (1.4 microgram/L).

The patient’s remaining hospital course was complicated by acute-onset dysuria and incontinence secondary to a Morganella morganii lower urinary tract infection (UTI), which was treated with a three-day course of ceftriaxone. The patient was successfully discharged to homecare on hospital day 10, with a plan for a tentative elective revision of the right hip prosthesis. Additionally, he was prescribed a five-day course of oral cefpodoxime for his UTI. The patient is currently pending outpatient orthopedic follow-up.

## Discussion

Despite a peak in popularity at the beginning of the 21st century, MoM THAs have been associated with an elevated risk for implant failure and the development of ARMD ranging from hip tenderness and swelling to localized tissue inflammation, metallosis, edema, systemic chromium and cobalt toxicity, and the development of pseudotumor. Post-MoM pseudotumor refers to the formation of a cystic lesion at the site of the original THA and is theorized to be an acute inflammatory response to metallic debris, causing lymphocyte recruitment with subsequent soft tissue fibrosis and necrosis [11]. Despite often being asymptomatic, large cysts may cause a nonspecific prodrome, including palpable hip mass, periprosthetic tenderness, and a mass effect [12]. Additionally, some patients may experience a rise in serum chromium and cobalt levels in their blood, urine, and/or synovial fluid due to prosthetic leakage, although it is not always seen. The incidence of pseudotumor is believed to be as high as 69%, with reported risk factors including female gender, a cobalt level >5 micrograms/liter, implant tenderness, and an inclination angle >55 degrees [11,13].

We report a unique case of an elderly man who presented nearly two decades following his index MoM-THA with complaints of right leg tenderness and poor weight-bearing and ambulation. Differential diagnoses at admission included a new hip fracture, which was promptly ruled out on standard radiographs, PJI, and bursitis. The patient did not have systemic symptoms or inflammatory markers, making PJI less likely, and was originally believed to have bursitis of the right lesser trochanteric bursa per CT imaging. However, when the patient’s pain persisted despite pain medication, an MRI was ordered, which showed two large pseudotumor cysts at the level of the ischial tuberosity and superior acetabulum, respectively, with subsequent sciatica nerve impingement. The patient was managed with ultrasound-guided drainage of the hip and pain medication, with a plan for future revisions of THA. Antibiotics were held due to the low risk of PJI, and no chelator therapy was needed as the patient’s chromium and cobalt came back within range.

Previously, in 2018, Grote and colleagues [7] described the management of a pseudotumor from MoM THA in a 58-year-old female with unilateral leg edema resulting from a mass effect on the external iliac vein. After ruling out deep vein thrombosis (DVT), the patient successfully underwent revision MoP THA and excision of the cyst, with complete resolution of her symptoms at a four-year follow-up. Unlike our case, the patient presented with elevated cobalt (15 micrograms/mL) and chromium metal ions in her blood (14 micrograms/mL), although she was not medicated with chelators. Another case published in 2016 by Leung et al. [14] described the complex management of MoM THA pseudotumor causing femoral nerve impingement in a 53-year-old man six years after an index operation. Despite the revision of THA to a ceramic-on-polyethylene implant, the patient’s neuropathy continued to worsen over time, and a repeat MRI revealed a growth in the cyst despite the removal of the MoM implant. The patient later underwent a laparoscopy excision of the retroperitoneal pseudotumor and recovered all his femoral motor functions with residual sensory deficits at a 17-month follow-up.

Notably, while many centers have discussed the presentation, workup, and management of patients presenting with pseudotumor or metallosis following MoM THA, pseudotumor-induced sciatica, to our knowledge, has only previously been described once in a 1999 case report by Fischer et al. [15]. In that study, an 84-year-old woman presented seven years after THA due to pain in her left hip and a grinding sensation when ambulating. CT revealed a large cyst occupying the greater sciatic notch and compressing the sciatic nerve. Under CT guidance, the authors successfully performed a percutaneous drainage of the cyst, followed by orthopedic surgical revision.

One potential complication of MoM THA that we did not encounter in our patient is systemic cobalt/chromium toxicity, which can arise due to leakage from the MoM implant into the bloodstream. The pathomechanism of chromium-cobalt toxicity is not fully understood, but it is hypothesized that the ions induce oxidative stress within macrophages and inhibit enzymatic reactions along the electron transport chain [16]. Systemic side effects of chromium/cobalt intoxication include neuropathy of peripheral nerves, weight loss, fatigue, and hearing/vision loss in severe cases [17,18]. Many centers have reported success using N-acetyl-cysteine (NAC) as a chelating agent, which works by directly binding ions via the thiol group. Of note, a 2020 case report demonstrated that asymptomatic metal toxicity after MoM THA could be successfully treated with high-dose NAC chelation without surgical revision [19]. As further investigation is done into the therapeutic use of prophylactic NAC in patients with MoM THA, one major limitation of chelation therapy continues to be the inability to neutralize large amounts of metal ions found in joint spaces [20].

## Conclusions

Our report demonstrates a unique complication of MoM THA in an 84-year-old man who presented with right leg pain 19 years after his index operation and was found to have sciatic nerve impingement due to periprosthetic pseudotumor. Faced with a similar presentation, clinicians must be meticulous in their musculoskeletal workup and use multimodality imaging to identify pseudotumor size, location, and adjacent neurovasculature to guide their management. Consideration of implant-related complications should be of high suspicion. Further, systemic metal ion toxicity should be ruled out or managed with NAC chelator therapy, and multidisciplinary collaboration should be embraced early to manage patient symptoms, avoid further tissue damage, and improve patient functionality. Finally, while large pseudotumors can be drained and cultured, definitive treatment will often require revision THA with an alternative bearing surface, such as metal-on-cross-linked polyethylene implants.
